# Mercury-Induced Oxidative Stress Response in Benthic Foraminifera: An In Vivo Experiment on *Amphistegina lessonii*

**DOI:** 10.3390/biology11070960

**Published:** 2022-06-24

**Authors:** Caterina Ciacci, Michele Betti, Sigal Abramovich, Marco Cavaliere, Fabrizio Frontalini

**Affiliations:** 1 Department of Biomolecular Science, Urbino University, 61029 Urbino, Italy; michele.betti@uniurb.it; 2 Department of Earth and Environmental Sciences, Ben Gurion University of the Negev, Beer Sheva 8410501, Israel; sigalabr@bgu.ac.il; 3 Department of Pure and Applied Sciences, Urbino University, 61029 Urbino, Italy; m.cavaliere6@campus.uniurb.it (M.C.); fabrizio.frontalini@uniurb.it (F.F.)

**Keywords:** benthic foraminifera, oxidative stress, protein, heavy metals

## Abstract

**Simple Summary:**

Mercury, one of the most hazardous metals, is known to pass through cellular membranes, bioaccumulate, and biomagnify. The impact of pollutants on marine environments has been evaluated mostly through bioindicators, but the recent advances in ‘omic’ technologies have opened a new opportunity for understanding, at the molecular level, the effects of pollutants such as mercury on biota. In the present research, physiological changes after mercury exposure in the symbiont-bearing benthic foraminiferal species *Amphistegina lessonii* are evaluated using several biomarkers (i.e., proteins and enzymes). Mercury leads to significant changes in the biochemistry of cells. Its effects are mainly associated with oxidative stress, depletion of glutathione, and alteration of protein synthesis. Overall, the observed biochemical alterations associated with mercury exposure may prove to be effective and reliable proxies (i.e., biomarkers) for the evaluation of stress in *A. lessonii* and may be useful in the detection of early warning signs of environmental stress in biomonitoring.

**Abstract:**

The evaluation of the effects of pollution (e.g., Hg pollution) is a difficult task and relies mostly on biomonitoring based on bioindicators. The application of biomarkers may represent a complementary or alternative approach in environmental biomonitoring. Mercury is known to pose a significant health hazard due to its ability to cross cellular membranes, bioaccumulate, and biomagnify. In the present research, the effects of short-term (i.e., 24 h) Hg exposure in the symbiont-bearing benthic foraminiferal species *Amphistegina lessonii* are evaluated using several biomarkers (i.e., proteins and enzymes). Mercury leads to significant changes in the biochemistry of cells. Its effects are mainly associated with oxidative stress (i.e., production of reactive oxygen species: ROS), depletion of glutathione (GSH), and alteration of protein synthesis. Specifically, our findings reveal that exposure to Hg leads to the consumption of GSH by GPx and GST for the scavenging of ROS and the activation of antioxidant-related enzymes, including SOD and GSH-enzymes (GST, GSR, GPx, and Se-GPx), that are directly related to a defense mechanism against ROS. The Hg exposure also activates the MAPK (e.g., p-p38) and HSP (e.g., HSP 70) pathways. The observed biochemical alterations associated with Hg exposure may represent effective and reliable proxies (i.e., biomarkers) for the evaluation of stress in *A. lessonii* and lead to a possible application for the detection of early warning signs of environmental stress in biomonitoring.

## 1. Introduction

Heavy metals arise from both natural origins (i.e., volcanoes, fires, etc.) and human-related activities [1]. Some heavy metals are micronutrients; these are essential to life and involved in various biological and physiological processes. Others (e.g., Hg, Cd) are non-essential and are toxic to living organisms at higher concentrations [2]. Mercury (Hg), one of the most hazardous metals, is known to pass through cellular membranes, bioaccumulate, and biomagnify [3,4]. In the open ocean and coastal seawater, dissolved Hg concentrations range from 0.5 to 3.0 ng/L and from 2.0 to 15 ng/L, respectively [1].

The impact of pollutants such as Hg on marine environments has been mostly evaluated through bioindicators [5]. The recent advances in omic technologies (e.g., genomics, proteomics, metabolomics, and transcriptomics) have, however, provided new tools for understanding, at the molecular level, the effects of pollutants on biota [6]. The molecular, biochemical, cellular, and physiological alterations (i.e., biomarkers) in a biological system can be used to detect the toxic effects of pollutants and can eventually be applied in environmental biomonitoring [7], even as an early warning of stress [8]. Moreover, protein biomarkers can be used to track and quantify individual pollutants [9]. Despite this, only a limited number of studies have been performed to assess water pollution through biomarkers [9].

The production of reactive oxygen species (ROS) by various biochemical mechanisms in mitochondria and peroxisomes is an important component of cellular respiration. However, the production of ROS is also an organism’s physiological response to a variety of stressors including pollutants [10]. Indeed, the metabolism of xenobiotics (e.g., Hg) leads to a decrease in the activity of the enzymatic antioxidant defense system and, ultimately, to oxidative damage [11]. The primary cellular antioxidants are enzymes such as superoxide dismutase (SOD), catalase (CAT), glutathione peroxidase (GPx), glutathione S-transferase (GST), and glutathione reductase (GSR) [12], as well as non-enzyme antioxidants such as vitamins and glutathione (GSH) that protect the cell by directly scavenging superoxide radicals (•O_2_^−^) and hydrogen peroxide (H_2_O_2_) and converting intracellular ROS to less-reactive species. Additional biomarkers include proteins such as metallothioneins (MT), heat-shock proteins (HSPs), mitogen-activated protein kinase (MAPK), and protein kinase C (PKC) [13,14,15]. Proteins, being the molecular effectors of biological processes, are suitable candidate biomarkers because their modulation is likely to be associated with the impact of one or more pollutants on an organism’s physiology [16].

Benthic foraminifera, marine protozoa, have been largely used as pollution bioindicators in marine and transitional marine environments [17]. However, only a few investigations have focused on the physiological and ultrastructural alterations induced by metals [18,19,20], and even fewer have examined their biochemical (i.e., biomarker) alterations [21,22,23,24]. Biochemical (e.g., protein or enzyme) changes in foraminifera resulting from stressing factors (e.g., heavy metals) can provide valuable information on the marine environmental quality [25]. Indeed, the development of cellular biomarkers might represent a complementary approach to traditional biomonitoring [23]. Increases in total SOD activity, lipid peroxidation, and MT-like protein content in *Amphistegina lessonii* were shown to result from Zn exposure [24]. Similarly, the inhibition of enzymatic activity (Ca^2+^-ATPase) in *Amphistegina gibbosa* was found to be related to variations in pH and Cu [21,22]. Decreased expression of the ribulose 1-5-bisphosphate carboxylase/oxygenase (RuBisCO) protein in the *Baculogypsina sphaerulata* holobiont was shown to be associated with increased temperature (i.e., +8 °C at 34 °C), whereas enhanced production of HSP 70 was documented in *Ammonia tepida* when exposed to different temperatures [26].

*Amphistegina* is a larger symbiont-bearing benthic foraminiferal genus that is particularly abundant on warm tropical and sub-tropical coral reefs and tropical carbonate shelves [27]. The genus has been reported to play a major role as an ecosystem engineer by significantly contributing to carbonate production [28]. Its distribution is mostly controlled by temperature [29], water transparency, and, indirectly, water depth. This genus has experienced a rapid expansion of its biogeographic range toward higher latitudes, approximately 11.76–13.82. km per year in the Mediterranean Sea [28,30]. This has resulted in direct consequences on indigenous species, restructuring communities, ecosystem functioning, and carbonate production [27]. *Amphistegina lobifera, Amphistegina lessonii,* and *Amphistegina* cf. *papillosa* have been recorded in the Mediterranean Sea [31]. *Amphistegina* species have been suggested to be sensitive to environmental stress (e.g., excess solar light) [32]. They have been used for the calculation of the FoRAM Index, a metric for coral reef water quality [33,34], and in various other laboratory experiments [35]. *Amphistegina lessonii* has also been used as a bioindicator of water quality in the Fernando de Noronha Archipelago in Brazil [23].

The main aim of the present study is to document the short-term effects of Hg exposure in *A. lessonii* using several biomarkers (i.e., proteins and enzymes) to detect the oxidative stress response at the cellular level. Several experiments have demonstrated the detrimental effect of Hg on benthic foraminiferal species [36]. Hg exposure has been reported to induce morphological abnormality, reductions (i.e., dwarfism) and delays in growth, intracellular lipid accumulation, lysosomal compartment amplification, cytoplasmic degradation, increased electron density core in lipids, and degraded mitochondria [20,37,38,39].

## 2. Materials and Methods

### 2.1. Individual Collection and Experimental Setup

Individuals of *A. lessonii* were collected from rock pebbles at approximately 1–2 m water depth at Eilat (Gulf of Aqaba, Red Sea, Israel) in early July of 2021. The site of sampling was close to the Inter-University Institute for Marine Sciences and is known as a relatively clean environment [35]. Adult living individuals about 600 to 800 µm in size were placed in 50 mL tubes and delivered to Urbino University (Italy). Once in the laboratory, individuals were placed in 100 mm glass Petri dishes with natural seawater (NSW), with a salinity of 40.4 and a pH of 8.15, collected from the sampling site. They were subjected to two-week acclimatization at 25 °C with 12:12 h light and dark cycles. Living individuals with a golden-brown color showing pseudopodial activity were selected for the experiment.

Mercury chloride (HgCl_2_; >99.5% pure; CAS number 7487-94-7; Sigma-Aldrich, St. Louis, MO, USA) was used for the experiments. According to the U.S. national recommendations for aquatic life, the maximum acute and chronic concentrations for Hg in saltwater (for one hour and four days’ exposure, respectively) are 1.8 and 0.94 μg/L (ppb) [40]. The predicted no-effect concentration (PNEC) in marine water is not available for HgCl_2_, but only for Hg (EC number 231-106-7), for which it is set at 0.067 µg/L (ppb) [41]. A stock solution of 1 mg/L Hg^2+^ was prepared by dissolving HgCl_2_ in filtered (0.22 μm) NSW and subsequently diluting the solution to the nominal concentration (10 μg/L). The Hg concentration was selected based on environmental relevance, concentrations previously used in similar toxicity tests [42,43], and the avoidance of lethal effects. Individuals of *A. lessonii* were placed in glass Petri dishes with either no Hg (as a control) or Hg-spiked NSW (10 μg/L) for 24 h in five replicates.

### 2.2. Electrophoresis and Western Blotting

Batches of 40 individuals were ground and homogenized with a homemade lysis buffer [25]. Specifically, samples were lysed with 1 mL of ice-cold lysis buffer (50 mM Tris-HCl, pH 7.8, 0.25 M sucrose, 1% (*w*/*v*) SDS (sodium dodecyl sulfate), 1 μg/mL pepstatin, 10 μg/mL leupeptin, 2 mM sodium orthovanadate, 10 mM NaF, 5 mM EDTA, 5 mM NEM (N-ethylmaleimide), 40 μg/mL PMSF (phenylmethylsulfonyl fluoride), 0.1% Nonidet-P40) and sonicated. Samples were boiled for 10 min and then centrifuged for 20 min at 14,000× *g* to remove insoluble debris. Supernatants were mixed 1:1 (*v*:*v*) with sample buffer (0.5 M Tris-HCl, pH 6.8, 2% SDS, 10% glycerol, 4% 2-mercaptoethanol, 0.05% bromophenol blue), and samples (normalized to 30 μg protein content before loading) were resolved either by 10% (for HSP) or 12% (for phospho-p38 MAPK) SDS-polyacrylamide gel electrophoresis [44]. Pre-stained molecular-mass markers were run on adjacent lanes. The gels were electroblotted on a nitrocellulose membrane [45]. Blots were then probed with the specific anti-phospho-p38 and anti-HSP (1:1000) as primary antibodies, and horseradish-peroxidase-conjugated goat anti-rabbit IgG (1:3000) was used as a secondary antibody. After antibody probing, nitrocellulose membranes were stripped for 30 min at 50 °C with stripping buffer (62.5 mM Tris–HCl, pH 6.7, containing 10 mM β-mercaptoethanol and 2% SDS) and re-probed with anti-actin (1:200). Western-blot films were digitized (Chemidoc-Biorad), and band optical densities were quantified using a computerized imaging system (Quantity One software) that also calibrated the molecular weights of the sample bands using both LWM and HMW standards. Relative optical densities (arbitrary units) were normalized for the control band in each series.

### 2.3. Antioxidant Activity Assays

#### 2.3.1. Total Superoxide Dismutase Assay

Total superoxide dismutase (SOD) activity was measured following the method described by Prazeres et al. [24] with some modifications. This method is based on the xanthine/xanthine oxidase assay and the reduction of cytochrome c [46,47]. The assay was carried out with foraminiferal homogenates (40 individuals in 300 µL of reaction buffer) containing potassium phosphate (KH_2_PO_4_, 50 mM), cytochrome c (10 μM), xanthine oxidase (50 μM), and EDTA (100 μM) with a solution pH of 7.8. The homogenate was sonicated and centrifuged for 10 min at 13,000× *g*. The reaction began with the addition of 0.5 U of xanthine oxidase in a final volume of 1000 μL of the reaction mixture. Cytochrome *c* reduction was observed spectrophotometrically (550 nm) for 5 min. Results were expressed as units of SOD activity per milligram of protein (U/mg of protein), where 1 unit of SOD is defined as the activity causing 50% inhibition of cytochrome c reduction at 25 °C.

#### 2.3.2. Reduced Glutathione Assay

Reduced (GSH) and oxidized (GSSG) glutathione were measured using the method described by Akerboom and Sies [48]. This method is based on the reduction of GSSG to GSH in the presence of glutathione reductase and NADPH and the formation of the colored product by the reaction of GSH with DTNB (5,5′-dithio-bis(2-nitrobenzoic acid)), followed by its absorbance at 412 nm at 25 °C. A total of 40 individuals were homogenized in 4 vol. of 1 N perchloric acid, 2 mM EDTA, then centrifuged at 16,000× *g* for 20 min at 4 °C. The supernatants were neutralized with 2 M KOH, 0.3 M morpholin ethanol sulphonic acid (MOPS) and centrifuged at 1000× *g* for 10 min at 4 °C. Aliquots of the neutralized supernatant were utilized for the evaluation of the total GSH concentration (i.e., molar sum of GSH + ½ GSSG). This was done using the enzymatic GSSG reductase assay in 1 mL of a reaction mixture containing 0.1 M K-phosphate buffer, 1 mM EDTA, pH 7.0, 0.2 mM NADPH, 0.06 mM dithionitrobenzoic acid, and 0.12 U of GSSG reductase. Calibration was performed utilizing known concentrations of GSSG instead of samples.

#### 2.3.3. Glutathione-Related Enzymes Assays

GST, GPx, selenium-glutathione peroxidase (Se-GPx), and oxidized glutathione reductase (GSR) activities were evaluated as previously described [49]. A total of 40 individuals were homogenized in 300 µL of 20 mM Tris–HCl buffer, pH 7.6, containing 0.5 M sucrose, 0.15 M NaCl, and then centrifuged at 20,000× *g* for 90 min at 0–4 °C. Aliquots of the supernatants were utilized for the spectrophotometric determination of enzyme activities. GST activity was evaluated with CDNB (1-chloro-2,4-dinitrobenzene) as a substrate. The reaction mixture (1 mL) contained 125 mM K-phosphate buffer, pH 6.5, 1 mM CDNB, and 1 mM GSH. The formation of S-2,4-dinitrophenyl glutathione conjugate was evaluated by monitoring the increase in absorbance at 340 nm. GSR activity was estimated in 1 mL of a reaction mixture containing 125 mM K-phosphate buffer, pH 7.5, 0.05 mM NADPH, and 1 mM GSH. The NADPH oxidation was evaluated by monitoring the increase in absorbance at 340 nm. GPx catalyzes the oxidation of glutathione by cumene hydroperoxide. In the presence of GSR and NADPH, the oxidized form of glutathione is immediately converted to the reduced form with concomitant oxidation of NADPH to NADP+. The decrease in absorbance at 340 nm was measured. The Se-GPx activity was measured using H_2_O_2_ as the substrate for the oxidation of GSH. The molar extinction coefficients (ε) were set at 9.6 µmol/cm for GST and 6.2 µmol/cm for GSR, GPx, and Se-GPx.

### 2.4. Statistical Analysis

Differences between control and treatment samples in the mean values of enzymes (i.e., SOD, GST, GSR, GPx, and Se-GPx) and proteins (i.e., HSP 70 and Phospho-p38) were analyzed by one-way analysis of variance (ANOVA). Data were square-root transformed to meet ANOVA assumptions. Post hoc analysis was performed using Tukey’s honestly significant difference (HSD) tests. The confidence level was set at 99% (α = 0.01).

## 3. Results

### 3.1. Glutathione and Enzymatic Biomarkers

**Figure 1:**
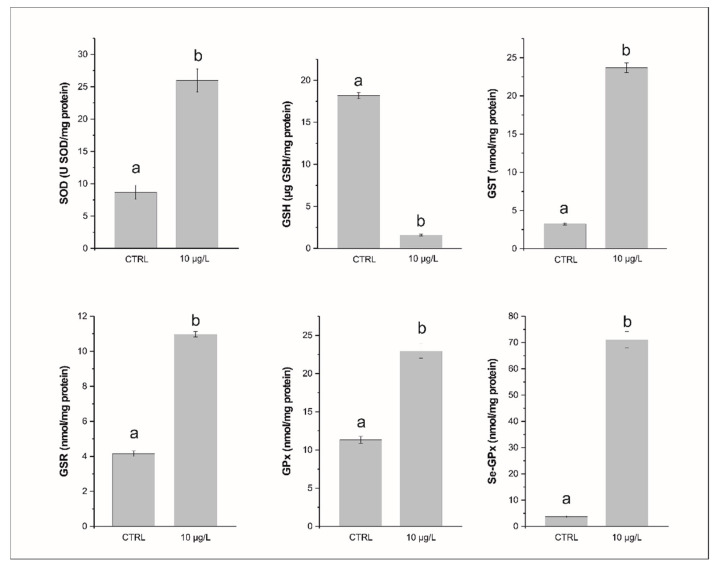
Levels of total superoxide dismutase (SOD), glutathione (GSH), the glutathione S-transferase (GST), glutathione reductase (GSR), glutathione peroxidase (GPx), and selenium-glutathione peroxidase (Se-GPx) in the symbiont-bearing foraminiferal species *Amphistegina lessonii* in samples exposed for 24 h at 10 µg/L of Hg and in control samples (no added Hg). Data are reported as mean ± standard deviation (*n* = 5). Letters denote significant differences (*p* < 0.01) between groups.

The SOD activity showed significantly higher (*p* < 0.0001) values in treatment samples (26.00 ± 3.50 U SOD/mg protein) than in control samples (8.65 ± 2.08 U SOD/mg protein; Figure 1 and Table S1). The GSH levels in the treatment samples (1.64 ± 0.15 GSH/mg protein) were significantly lower (*p* < 0.0001) than in controls, which averaged 18.19 ± 0.74 µg GSH/mg protein (Figure 1 and Table S1). The GST exhibited significantly higher (*p* < 0.0001) values in treated samples (23.70 ± 1.17 nmol/mg protein) than in control samples (3.25 ± 0.24 nmol/mg protein; Figure 1 and Table S1). The GSR levels were significantly higher (*p* < 0.001) in treated samples (10.98 ± 0.27 nmol/mg protein) than those in control samples (4.16 ± 0.31 nmol/mg protein; Figure 1 and Table S1). The GPx exhibited significantly higher (*p* < 0.0001) levels in treated samples (22.92 ± 1.81 nmol/mg protein) than in control samples (11.34 ± 1.04 nmol/mg protein; Figure 1 and Table S1). The Se-GPx values are significantly higher (*p* < 0.0001) in treated samples (71.07 ± 5.62 nmol/mg protein) than in controls (3.71 ± 0.60 nmol/mg protein; Figure 1 and Table S1).

### 3.2. Effects on p38 MAPK Phosphorylation and HSP 70

**Figure 2:**
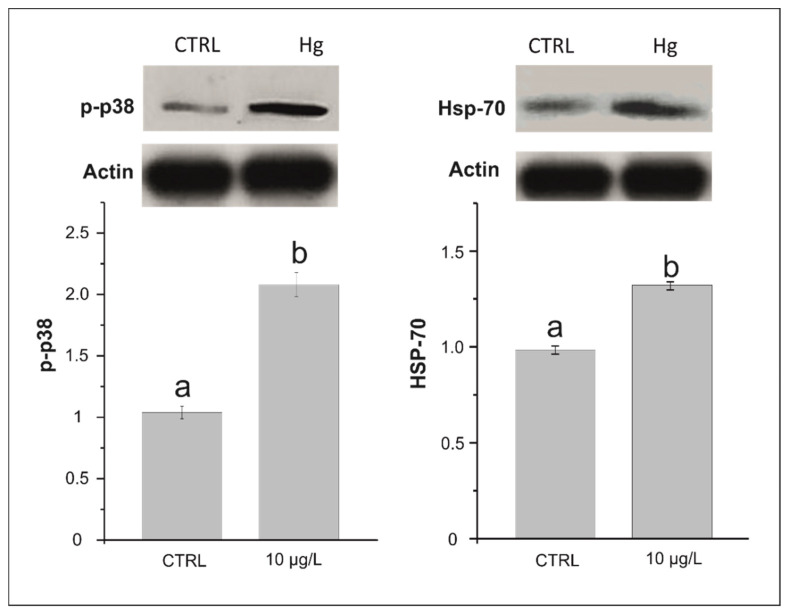
Levels of HSP-70 and p-p38 in the symbiont-bearing foraminiferal species *Amphistegina lessonii* in samples exposed for 24 h at 10 µg/L of Hg and control samples (no added Hg). Data are reported as mean ± standard deviation (*n* = 5). Letters denote significant differences (*p* < 0.01) between groups.

The amount of detected p-p38 was significantly higher (*p* < 0.0001) in treatment samples (2.07 ± 0.19) than in control samples (1.04 ± 0.09; Figure 2 and Table S2). Similarly, the HSP 70 content was significantly higher (*p* < 0.0001) after treatment (1.33 ± 0.05) compared with that in control samples (0.98 ± 0.05; Figure 2 and Table S2).

## 4. Discussion

In the present research, the short-term effects (i.e., 24 h) of Hg exposure in *A. lessonii* were evaluated using several biomarkers (i.e., proteins and enzymes). Heavy metal ions including Hg are known to induce ROS generation and cellular damage [50]. In fact, exposure to organic (methylmercury, MeHg+) or inorganic (mercury chloride, HgCl_2_) Hg leads to significant changes in the biochemistry of cells. Its effects are mainly associated with oxidative stress, depletion of GSH, and altered protein synthesis [51,52]. Mitochondria function as the most important ROS-generation sites in the cell, and their disruption or functional alteration might lead to an imbalance of ROS scavenging and RNA leakage that ultimately results in intracellular oxidative stress [53]. Hg promotes the inactivation of enzymes containing thiol- (-SH) and seleno- (-SeH) groups as well as proteins responsible for antioxidant defense [54,55]. It has been reported that HgCl_2_ may be a relevant apoptosis inducer through cytochrome c release and the activation of p38 MAPK [51]. The primary mechanism of Hg toxicity lies in its ability to covalently bind with GSH, resulting in the overall depletion of the mitochondrial GSH pool once excreted from the cell [52,55,56]. Accordingly, a single Hg ion can account for the irreversible excretion of up to two molecules of GSH [56,57]. Antioxidant activity is also impaired after Hg treatment, though the response of each enzyme greatly varies with the experimental conditions [58]. Accordingly, several experiments have revealed that Hg alters the activity of enzymatic antioxidants, namely lipid hydroperoxide (LPO), GSR, GPx, SOD, and catalase (CAT), among others, and non-enzymatic antioxidants such as GSH and vitamins C and E [59,60,61]. Similarly, in our study, an increase in antioxidant enzymatic activity was associated with Hg treatment for all tested enzymes (i.e., SOD, GST, GSR, GPX, and Se-GPX). Specifically, significantly higher concentrations of SOD were associated with Hg treatment. A similar pattern has been found in pale/partly bleached individuals of *A. lessonii* when exposed, under laboratory-controlled conditions, to increasing concentrations of zinc for 48 h, while this was not observed with normal-appearing individuals [23]. The authors attributed the SOD inhibition in normal-appearing individuals to severe oxidative stress that induced inactivation and degradation of this enzyme. One of the most important antioxidant components is SOD, which acts as a first-line ROS scavenging enzyme [62]. SOD is widely found in prokaryotes and eukaryotes and catalyzes the dismutation of superoxide ion (•O_2_^−^) into hydrogen peroxide (H_2_O_2_) and molecular oxygen (O_2_) [63]. Enhanced SOD activity has been found in plants in response to water deficiency and saline or supplemental ultraviolet-B stress [63], as well as in fish living in extremely PCB-polluted conditions [64].

To the best of our knowledge, this research presents the first measurement of GSH in a foraminiferal species; therefore, data are not available for comparison. Our data indicate a significant reduction of GSH in the foraminiferal species *A. lessonii* after 24 h exposure to Hg. GSH is a tripeptide found in all eukaryotic and many prokaryotic cells [65].

In most eukaryotes, GSH is the most important intracellular non-protein thiol group and represents the first line of defense against metal ions [56]. It functions as an antioxidant by scavenging ROS (e.g., hydroxyl radicals), preventing oxidative stress, and acting as an essential substrate for GST, GR, and GPx [10,12]. The levels of GSH, however, decrease when excessive oxidative stress prevails within the cell [66], leading to the intracellular flux of radicals and further oxidative stress [67]. A low concentration of GSH may result in oxidation mediated by GPx or the formation of conjugates with GSH mediated by GST [68]. Low concentrations of Hg (e.g., 12 nmol Hg/mg protein) are reported to completely deplete mitochondrial GSH in 30 min and stimulate the generation of H_2_O_2_ in rat kidneys [69,70]. Similar results have been observed in different species, including protists (e.g., *Paramecium*), exposed to high concentrations of heavy metals [15,71], which matches well with the depletion of GSH in response to Hg exposure seen in the present study. Thus, the decreased GSH level in *A. lessonii* can be interpreted as an impairment of the GSH-related defense system that results in increased oxidative damage.

GSH-related enzymes (i.e., GST, GSR, GPx, and Se-GPx), to our knowledge, have not been studied in any foraminiferal research so far. Glutathione S-transferases (GSTs) play a major role in phase II detoxification by scavenging ROS. This enzyme can catalyze the conjugation of the reduced form of GSH to xenobiotics, converting them into less-toxic xenobiotics and ultimately leading to their removal from the cell by ATP-dependent efflux pumps [72,73]. This protein family has been identified in all life forms (i.e., animals, plants, protozoa, fungi, and bacteria) [63,74], and its expression is upregulated in a variety of organisms (e.g., bacteria, rotifer, copepods, and polychaete) when exposed to heavy metals (e.g., Hg, Cd, and Cu) [75]. Our data suggest a significant increase in GST in *A. lessonii* when exposed to Hg. Similar results have been found in copepods and rotifers after MeHg+ treatment [76,77].

Glutathione reductase (GSR) catalyzes the reduction of GSSG to GSH by NADPH [72]. This enzyme is also involved in the detoxification of xenobiotic compounds through GSH conjugation. The expression of GSR was found to be upregulated in different living organisms (e.g., ciliates, rotifers, zebrafish) after exposure to pesticides (e.g., diazinon and diuron) and heavy metals (e.g., Hg, Cd, Cu, Pb, Zn) [76,77,78,79]. Methylmercury treatment in copepods and rotifers was found to increase GSR activity [76,77]. Similarly, an increase in GSR was found in *A. lessonii* in response to Hg.

Glutathione peroxidase (GPx) is an enzyme that catalyzes the reduction of H_2_O_2_ and lipid hydroperoxides to water and lipid alcohols using GSH [80]. GPx-like proteins have been documented in all kingdoms of life [80,81]. Most metazoans possess selenium-dependent glutathione peroxidase (Se-GPx) activity [80]. The expression of several GPx-associated genes (e.g., GPx-1a and GPx-1b) was observed to be upregulated in several organisms after exposure to pesticides and heavy metals (e.g., Cd, Cu, Pb, Zn) [15,79]. Although a reduction in GPx activity was found in the rotifer species *Brachionus koreanus* after MeHg^+^ exposure, the mRNA expression level of GPx was upregulated [68]. Moreover, increased GPx activity was found in the copepod *Paracyclopina nana* in response to MeHg^+^ [77]. Our results fit well with these data by revealing increased activity of both GPx and Se-GPx in *A. lessonii* in response to Hg exposure.

It has been demonstrated that the oxidative stress caused by metal exposure (e.g., Hg) induces protein modification (e.g., MAPKs) and activation (e.g., HSPs) [15,82]. Mitogen-activated protein kinases (MAPKs) are a family of serine/threonine kinase signal transduction proteins involved in the regulation of several intracellular activities, programmed cell death, responses to stimuli, and adaptation to environmental modification (i.e., environmental stress) [83]. The MAPK cascade is an ancient and evolutionarily conserved signaling pathway. MAPKs are ubiquitously expressed in eukaryotes, and their function is the amplification of a signal by sequential phosphorylation events, a system that is sensitive to subtle changes in the cell environment [84]. A subfamily of MAPK, p-p38s, has been reported to play different roles (e.g., cellular differentiation), including metal-induced cell death [85,86]. Mercury chloride is known to activate the MAPK pathway via the generation of ROS [51], and different studies have revealed increased expression of p-p38 in response to HgCl_2_ treatment [87] that ultimately mediates apoptosis [88,89]. Similarly, our data suggest a significant increase of p-p38 in foraminifera (i.e., *A. lessonii*) when exposed to HgCl_2_.

Heat shock proteins (HSPs) are molecular chaperones found in a variety of cells in both eukaryotic and prokaryotic organisms of variable molecular weight (i.e., 10 to 174 kDa) [90,91,92]. HSPs are expressed under normal cellular conditions but show significant response when cells are exposed to various environmental stresses such as temperature variation, heavy metals, salinity, etc. [93]. HSPs mediate a wide range of cellular functions including protein folding, assembly, and repair, and their synthesis is enhanced under stress conditions (e.g., temperature, hypoxia, osmotic shock, and toxic substances). They are therefore known as ‘stress proteins’ [90,94]. HSP 70 and HSP 90 play a major role in protein homeostasis against metal-induced oxidative stress using cytoprotective effects [95] and can minimize protein aggregation. HSP 70 has been inferred to inhibit apoptosis as well as stimulate cytokine production and autophagy. HSPs have been identified in a wide array of marine organisms [15]. Increased synthesis of HSP 70 in chick embryos, as well as rat liver and kidney tissue, has been linked to Hg exposure [96]. This has also been observed in foraminifera in response to thermal stress [26]. Our data point to an increase in HSP 70 in *A. lessonii* after Hg exposure, which aligns with previous findings.

## 5. Conclusions

The short-term exposure of the symbiont-bearing foraminiferal species *A. lessonii* to Hg, a cytotoxic element, induces the activation of various non-enzymatic antioxidants, enzymatic antioxidants, and protein pathways. Our findings suggest that exposure to Hg leads to the consumption of GSH by GPx and GST for the scavenging of ROS (i.e., H_2_O_2_ and organic peroxides). Our data also indicate that the antioxidant-related activities of SOD and GSH enzymes (GST, GSR, GPx, and Se-GPx) are directly involved in a defense mechanism against ROS induced by Hg. Hg-induced oxidative stress activates the MAPK pathway and increases levels of p-p38. It also induces the activation of HSPs (e.g., HSP 70) for protein homeostasis regulation. Our results indicate the potential of using protists for discerning the relationships between relevant cellular mechanisms. Taken together, the observed biochemical alterations associated with Hg exposure may represent an effective and reliable proxy (i.e., biomarkers) for the evaluation of stress in *A. lessonii* and may be useful in the detection of early warning signs of environmental stress. This study supports the application of foraminifera as a model in ecotoxicological studies.

## Data Availability

The data presented in this study are available in Tables S1 and S2.

## Supplementary Materials

The following supporting information can be downloaded at https://www.mdpi.com/article/10.3390/biology11070960/s1: Table S1: Raw data of total superoxide dismutase (SOD), glutathione (GSH), the glutathione S-transferase (GST), glutathione reductase (GSR), glutathione peroxidase (GPx), and selenium-glutathione peroxidase (Se-GPx) in the symbiont-bearing foraminiferal species *Amphistegina lessonii*; Table S2: Raw data of HSP-70 and p-p38 in the symbiont-bearing foraminiferal species *Amphistegina lessonii*.
